# The relationship among positive body image, body esteem, and eating attitude in Iranian population

**DOI:** 10.3389/fpsyg.2024.1304555

**Published:** 2024-02-16

**Authors:** Hamid Sharif-Nia, Erika Sivarajan Froelicher, Ozkan Gorgulu, Jason W. Osborne, Aleksandra Błachnio, Azadeh Rezazadeh Fazeli, Amir Hossein Goudarzian, Omolhoda Kaveh

**Affiliations:** ^1^Psychosomatic Research Center, Mazandaran University of Medical Sciences, Sari, Iran; ^2^Department of Nursing, Amol Faculty of Nursing and Midwifery, Mazandaran University of Medical Sciences, Sari, Iran; ^3^Department of Physiological Nursing, School of Nursing, University of California, Sand Francisco, San Francisco, CA, United States; ^4^Department of Epidemiology and Biostatistics, School of Medicine, University of California, Sand Francisco, San Francisco, CA, United States; ^5^Faculty of Medicine, Department of Biostatistics and Medical Information, Kirsehir Ahi Evran University, Kirsehir, Türkiye; ^6^Department of Statistics, Miami University, Oxford, OH, United States; ^7^Department of Psychology, Kazimierz Wielki University in Bydgoszcz, Bydgoszcz, Poland; ^8^Department of Psychology, Islamic Azad University, Tonekabon, Iran; ^9^Student Research Committee, Mazandaran University of Medical Sciences, Sari, Iran; ^10^School of Nursing and Midwifery, Tehran University of Medical Sciences, Tehran, Iran; ^11^Department of Nursing, Sari Faculty of Nursing and Midwifery, Mazandaran University of Medical Sciences, Sari, Iran

**Keywords:** positive body image, body esteem, eating attitude, Iran, adult, adolescent

## Abstract

**Background and aim:**

The correlation between eating attitudes, positive body image, and body esteem is a pivotal area of research that has garnered substantial attention in recent years, given its implications for both mental and physical well-being. The objective of this study was to examine the interplay between positive body image, body esteem, and eating attitudes within an Iranian population.

**Materials and methods:**

This study employed a cross-sectional study design and was conducted in the year 2022. A convenience sample of 752 participants residing in Tehran, Iran, was included in the study. The data collection tools were comprised of a demographic registration form, the Adolescence/Adults Scale (PBIAS), the Eating Attitudes Test (EAT), and the Body Esteem Scale (BES) as measurement instruments.

**Results:**

Mean age of participants was 26.36 (SD = 8.49). Significant relationships were found among positive body image (*B* = − 0.095, *β* = −0.150, *p* < 0.001), and body esteem (*B* = 0.175, *β* = 0.149, *p* < 0.001) with eating attitudes.

**Conclusion:**

These findings suggest that individuals with positive body image and high body esteem may have healthier eating attitudes, while those with negative body image and low body esteem may be more likely to have unhealthy eating attitudes.

## Introduction

In the complex tapestry of human behavior and psychology, one of the most fundamental and essential aspects of daily life is eating (Hayes et al., 2018). Beyond its biological necessity, eating encompasses a rich interplay of attitudes, beliefs, and behaviors that reflect the intricate relationship between individuals and their sustenance. The study of eating attitudes delves into the various ways in which individuals perceive, approach, and engage with food. These attitudes are shaped by a myriad of factors, including cultural norms, societal influences, personal experiences, and psychological predispositions (Hayes et al., 2018; Aparicio-Martinez et al., 2019). Understanding eating attitudes is not only crucial for unraveling the mysteries of human behavior but also holds immense significance in addressing widespread concerns about eating disorders, obesity, and disordered eating patterns. The intricate connection between emotional states, mental health, and eating attitudes underscores the need for comprehensive research and exploration into this multifaceted domain (Tadena et al., 2020; Gürbüzer et al., 2023). This exploration involves delving into various dimensions, ranging from the development of attitudes about eating during different life stages, to the impact of media and social influences on shaping perceptions about body image and dietary choices. Additionally, the examination of cultural variations in eating attitudes sheds light on how different societies value and interact with food, reflecting on both shared traditions and evolving trends (Tayfur and Evrensel, 2020; Tatlibal, 2022).

The issue of eating attitudes is crucial in Iran due to a confluence of cultural, social, and health-related factors that shape the way individuals in our country perceive and interact with food (Tatlibal, 2022). Iran, like many other societies, has its unique set of challenges and dynamics that underscore the importance of addressing eating attitudes within its cultural context. In Iranian culture, food holds deep cultural and social significance. Traditional Persian cuisine is celebrated for its rich flavors and historical roots (Momeni et al., 2020). However, societal norms and expectations surrounding food can sometimes contribute to complex attitudes and behaviors (Asl et al., 2021). Balancing the preservation of cultural culinary heritage with the promotion of healthy eating attitudes can be a very well important aspect of health care. Like many places around the world, Iran grapples with societal pressure to conform to certain beauty standards, which can contribute to negative body image and unhealthy eating behaviors (Rouzitalab et al., 2019). The influence of media and social media platforms in promoting unrealistic body ideals can exacerbate these issues (Tayefi et al., 2020). Iran has experienced significant societal changes in recent decades, including shifts from traditional dietary patterns to more modern and processed food consumption. This transition has led to a shifts in eating attitudes and behaviors, potentially impacting health outcomes (Tajik and Lotfi Kashani, 2019; Tayefi et al., 2020). As Iran’s population becomes more urbanized and sedentary, concerns related to obesity, disordered eating, and eating disorders have grown. These issues not only have direct health implications but also highlight the importance of understanding and addressing eating attitudes as a means of promoting better overall health (Khoshkerdar and Raeisi, 2020). In some cases, seeking professional help for eating disorders (or unhealthy eating behavior) or related issues might be stigmatized due to cultural beliefs. Creating an environment where individuals feel comfortable seeking support is a challenge that needs to be addressed (Tajik and Lotfi Kashani, 2019; Khoshkerdar and Raeisi, 2020; Tayefi et al., 2020).

Based on the literature, factors that have a possible effect on eating attitudes, positive body image and body esteem attracted a lot of attentions. When individuals have a positive body image and high body esteem, they are more likely to engage in healthy eating attitudes (Costarelli et al., 2009). They view their bodies as valuable and worthy regardless of societal norms or unrealistic standards, which can lead to a greater focus on nourishing their bodies with balanced nutrition rather than engaging in restrictive or extreme dieting behaviors (Costarelli and Stamou, 2009). Positive body image is characterized by a realistic and healthy perception of one’s own body, along with self-acceptance and appreciation of its unique attributes. When individuals have a positive body image, they are more likely to feel comfortable and satisfied with their physical appearance (Gattario and Frisén, 2019). This positive view of their bodies can influence their attitudes toward food and eating. Body esteem goes beyond body image and refers to the overall sense of self-worth and self-esteem that an individual associates with their physical body. Having high body esteem involves feeling confident and valuable based on one’s body and its appearance (Pop et al., 2022). A positive body esteem can contribute to healthier eating attitudes by reducing the tendency to engage in harmful eating behaviors as a means of altering one’s body (Chang et al., 2019). Positive body image and body esteem can act as protective factors against the development of disordered eating attitudes. Conversely, individuals with negative body image and low body esteem are at a higher risk of developing unhealthy eating attitudes (Filaire et al., 2007; Costarelli and Stamou, 2009). The dissatisfaction with their bodies can lead to attempts to control their weight through extreme dieting, binge eating, or other disordered behaviors (Filaire et al., 2007). These behaviors are often driven by the desire to attain a certain body ideal and can result in negative emotional and psychological outcomes. It is important to note that these relationships are not unidirectional. Positive body image and body esteem can influence eating attitudes, and at the same time, engaging in healthy eating behaviors and adopting a balanced approach to eating can also improve body image and body esteem (Rouveix et al., 2007; Gitau et al., 2014). This creates a cyclical process where positive reinforcement occurs between these factors. Understanding the complex interplay between positive body image, body esteem, and eating attitudes is crucial for designing interventions and strategies that promote healthy eating behaviors, positive body image, and overall well-being (Petisco-Rodríguez et al., 2020). Approaches that aim to enhance self-acceptance, challenge unrealistic beauty standards, and encourage balanced eating can contribute to a more positive relationship with food and one’s body (Aparicio-Martinez et al., 2019).

The relationship between eating attitudes, positive body image, and body esteem is a crucial area of study that has gained significant attention in recent years due to its implications for mental and physical well-being (Tayfur and Evrensel, 2020). However, in the specific context of Iran, there remains a notable lack of comprehensive research that addresses this complex relationship. Studying body image and eating attitudes involves sensitive cultural considerations, as these topics intersect with traditional beliefs, cultural norms, and societal expectations (Momeni et al., 2020; Rostampour et al., 2022). Researchers may hesitate to delve into these topics due to potential cultural sensitivities or concerns about stigmatization (Momeni et al., 2020). To date, no comprehensive studies have been conducted in Iran addressing this issue with a sufficient sample size and diverse age groups. Consequently, this study was undertaken with the objective of evaluating the relationships among positive body image, body esteem, and eating attitudes within the Iranian population.

## Materials and methods

### Design and participants

This study employed a cross-sectional study design and was carried out in 2022. The study population consisted of individuals residing in Tehran, Iran, and they were recruited for participation in this research. A convenience sampling method was employed, and participants were required to meet specific criteria, including: (i) proficiency in reading Farsi; (ii) abstention from drugs; (iii) abstention from alcohol; (iv) the absence of advanced psychological disorders such as major depression or schizophrenia; (v) age under 65 years old; and (vi) access to the internet and social media. Individuals with major depressive disorders (assessed with self-reporting) were excluded due to the significant likelihood that depression could impact body image. The sample size was determined using G*Power 3.1.7 software, with the following assumptions: a two-tailed significance level (α) of 0.05, a power of 80%, and an effect size (d) of 0.2. The estimated sample size for the study was 752 individuals.

A Telegram (a social media network) group related to psychological topics was utilized. With this approach, the study’s objectives were initially communicated to the audience, and the responsible researchers were introduced. Subsequently, individuals expressed their willingness to participate and provided initial information such as age, and participants meeting the entry criteria were selected. In the next step, individuals above 18 years of age completed an online consent form, and then the link to the questionnaire content was sent to them. For individuals under 18 years of age, after obtaining consent from both the individual and their parents, a link containing the questionnaire was sent to the parents for the desired sample to complete. Necessary explanations were also provided to individuals before completing the questionnaire.

### Instruments

The data collection tools comprised a demographic registration form, the Positive Body Image among Adolescence/Adults Scale (PBIAS), the Eating Attitudes Test (EAT-26), and the Body Esteem Scale (BES). Also, demographic variables such as age, sex, height, and weight (used to compute body mass index) and other data such as social media usage (using Telegram, WhatsApp, or Instagram), and satisfaction with owns sex were gathered with a checklist.

#### Positive body image among adolescence/adults scale

The original version of PBIAS was conceptualized and developed by Maes et al. (2021). The scale was developed to assess the components of positive body image. It has 15 items with scoring by a Likert scale (1 = Strongly disagree to 7 = Strongly agree). Although this scale was developed for adolescents, it can also be useful for measuring body image in adults. This scale focuses more on the positive dimension of body image than the other available scales. Also, this scale is a comprehensive measure that evaluates different components of positive body image and can reflect the multidimensionality of positive body image. The psychometric equivalence of a construct across groups estimate through measurement invariance and proves that a construct is meaningful in the same way by those groups or across different measurements (Putnick and Bornstein, 2016). In the present study, the reliability of the PBIAS was calculated to be *α* = 0.859.

#### Eating attitudes test

The original version of EAT was conceptualized and developed by Garner et al. (1982). EAT is a 26 item, self-report inventory that measures dieting behaviors, food preoccupation, anorexia, bulimia, and concerns about being overweight. Scoring the 26 items of the EAT-26 is according to the Likert scoring system (0 = Never to 3 = Always). Scores greater than 20 indicate a need for further investigation by a qualified professional. Low scores (below 20) can still be consistent with serious eating problems, as denial of symptoms can be a problem with eating disorders (Garner et al., 1982). The EAT has demonstrated concurrent and predictive validity as well as reliability (Garner et al., 1982). In the present study, the reliability of the EAT was calculated to be *α* = 0.769.

#### Body esteem scale

The original version of BES was conceptualized and developed by Mendelson et al. (2001). This scale was developed to assess the components of body esteem. It has 23 items that are scored on a Likert scale (1 = never to 5 = always). This scale has been widely used as a self-report scale for body esteem. The original version of this scale includes three subscales: appearance (evaluation of general feelings and satisfaction with overall appearance), weight (evaluation of general feelings and satisfaction with weight), and attributes (evaluations attributed to others about an individual’s appearance) (Mendelson et al., 2001). This scale has been psychometrically tested in several populations and cultures, including Spanish (Beltrán-Garrayo et al., 2022), Italian (Confalonieri et al., 2008), and Turkish adolescents (Arslan et al., 2020), and its validity and reliability have been confirmed. In the present study, the three subscales (appearance, weight, and attribution) all correlated very strongly with the total score (*r* ranged from 0.55 to 0.90, all *p* < 0.001) and thus all items were summed to create a single BES total score. The reliability of the BES was calculated to be *α* = 0.877.

### Translation of scales

The scales were translated from English to Farsi following the translation protocol of Gudmundsson (2009). Two proficient English-Persian translators independently translated each scale into Farsi. An expert panel, comprised of some of the authors of this article and two professional translators, meticulously reviewed and amalgamated the two translations to create a Farsi version of the scale. Subsequently, a Persian-English translator was engaged to translate the Farsi version of the scale back into English. The panel of experts review and approved the final version.

### Data analysis

Data analysis was conducted using the Statistical Package for Social Sciences version 26.0 software for Windows (IBM SPSS Statistics for Windows, Version 26.0, Armonk, NY: IBM Corp., United States). Regression analyses were examined to ensure they met parametric assumptions.

The relationship between predictor variables and EAT was examined through several regression analyses. First, a multiple regression analysis predicting EAT from age and BMI was performed as these two variables had significant missing data (309 and 316 missing cases, respectively).

Second, other variables were entered as blocks of conceptually-grouped variables to reduce complexity. Background demographic variables (e.g., sex, family education and income, number of children in family, etc.) were entered in the first block. Next, we entered behavioral variables (e.g., social media usage, travel abroad, etc.). Finally, attitudes were entered as a block (e.g., BES, PBIAS) predicting EAT. The significance level for all tests was set at *α* < 0.05.

## Results

The demographic profile of the 752 participants is presented in Table 1. The mean age for adolescents was 16.55 (*n* = 252, SD = 1.5), while adults had a mean age of 30.66 (*n* = 500, SD = 7.40). The mean scores for positive body image, body esteem, and eating attitudes were as follows: positive body image (71.20, 95% CI: 70.27, 72.13), body esteem (73.87, 95% CI: 72.63, 75.11), and eating attitude (11.54, 95%CI: 10.59, 12.13).

**Table 1:** Demographic characteristics of the patients.

Variables	*N* (%) or Mean (SD)	Variables	*N* (%) or Mean (SD)
Sex		Occurrence of plastic surgery in the family and close relatives	
*Male*	659 (87.6%)	*Yes*	472 (62.8%)
*Female*	93 (12.4%)	*No*	280 (37.2%)
Father’s educational level		Presence of friends dissatisfied with their body appearance	
*Illiterate*	326 (43.4%)	*Yes*	72 (9.6%)
*High school*	62 (8.2%)	*No*	680 (90.4%)
*Diploma*	199 (26.5%)	Number of children in the family	
*Bachelor of sciences*	123 (16.4%)	*1*	287 (38.2%)
*Master of sciences and upper*	42 (5.6%)	*2*	197 (26.2%)
Mother’s educational level		*3*	121 (16.1%)
*Illiterate*	476 (63.3%)	*4*	108 (14.4%)
*High school*	77 (10.2%)	*5*	39 (5.2%)
*Diploma*	142 (18.9%)	Sex satisfaction	
*Bachelor of sciences*	42 (5.6%)	*Yes*	709 (94.3%)
*Master of sciences and upper*	15 (2.0%)	*No*	43 (5.7%)
Monthly family income		Social media usage	
*Weak*	84 (11.2%)	*Yes*	741 (98.5%)
*Average*	228 (30.3%)	*No*	11 (1.5%)
*Good*	219 (29.1%)	Age	26.36 (8.49)
*well*	221 (29.4%)	Height	164.44 (13.06)
		Weight	64.55 (15.13)

In regression analyses, presented in Table 2, six variables exhibited a significant impact on the prediction of eating attitudes. These variables included age, BMI, satisfaction with one’s sex, social media usage, positive body image, and body esteem.

**Table 2:** Variables predicting attitudes toward eating.

Predictor variables	Regression results				
	*B*	*β*	*t* value	*p* value	CI 95% for *B*
*Regression #1: R*^2^ = 0.028, *F_(2, 408)_ =* 5.87, *p* < 0.003					
Age, (year)	−0.14	−0.15	−2.81	**0.005**	−0.24, −0.04
Body mass index (BMI)	0.24	0.14	2.74	**0.006**	0.07, 0.42

*Regression #2, Block 1: R*^2^ = 0.01, *F_(7,744)_ =* 1.10, *p* < 0.36					
Sex, (F/M)	1.39	0.55	1.46	*ns*	
Father’s education level	−0.29	−0.05	−1.11	*ns*	
Mother’s education level	−0.13	−0.02	<1	*ns*	
Monthly family income	0.25	0.03	<1	*ns*	
Occurrence of plastic surgery in the family and close relatives, (No/Yes)	0.39	0.02	<1	*ns*	
Presence of friends dissatisfied with their body appearance, (Yes/No)	−0.29	−0.01	<1	*ns*	
Number of children in the family	−0.29	−0.04	<1	*ns*	
					
*Regression #2, Block 2: ΔR*^2^ = 0.034, *F_(5,739)_ =* 5.22, *p* < 0.001					
Sex satisfaction, (No/Yes)	−4.54	−0.13	−3.49	**<0.001**	−7.10, −1.99
Social media usage (No/Yes)	−4.11	−0.06	−1.59	*ns*	
Instagram usage (No/Yes)	−1.23	−0.04	<1	*ns*	
Time duration of social media usage	1.09	0.12	3.26	**0.001**	0.44,1.75
Having traveled abroad in the last 5 years	0.77	0.04	1.18	*ns*	
					
*Regression #2, Block 3: ΔR*^2^ = 0.13, *F_(2,737)_ =* 55.65, *p* < 0.001					
Positive body image (PBIAS)	0.088	0.14	3.07	**0.002**	0.03, 0.15
Body esteem scale (BES)	−0.21	−0.44	−9.73	**<0.001**	−0.25, −0.17

*B* represents the unstandardized regression coefficient. *β* reflects the standardized regression coefficient. Bold values refers to significance levels.

However, as you can see in Table 2, other common demographic and behavioral variables showed no unique predictive power individually or as a block. Biological sex, income, parent education, and other potential predictors were found not to be significant predictors. The largest effects were the two attitudinal variables, positive body image and Body self-esteem, which accounted for 13% of the variance in the outcome variable above after the variables in the first two blocks were accounted for.

Overall, these analyses show mostly small effects in predicting attitudes toward eating except for positive body image and body self-esteem. In simple univariate analyses, both of these variables were negatively correlated with attitudes toward eating. However, when entered together in the regression analysis, it was primarily Body Esteem that had the larger unique effect.

Given the literature on sex differences in these variables, we explored whether sex moderated the two primary attitudinal variables PBIAS and BES. We found no significant interaction terms.

### Nonlinear effects

**Figure 1:**
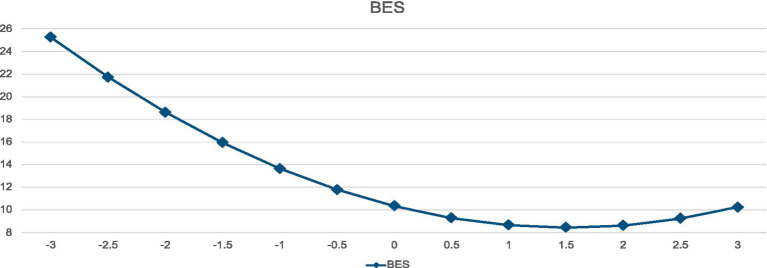
Curvilinear relationship between BES and EAT.

We also examined whether there were nonlinear relationships between EAT and BES or PBIAS. Following recommended practices, PBIAS and BES were centered by conversion to *z*-score distributions, and squared. Then, in separate analyses, the original variable was entered on the first step and the squared term on the second step to evaluate whether the nonlinear effect accounted for significant additional variance. As you can see in Table 3 and Figure 1, there was a modest nonlinear effect for BES, showing the most dramatic effects of BES on EAT in the lower range of the BES distribution, asymptoting as scores move toward 1–1.5 SD above the mean.

**Table 3:** Curvilinear effects of BES on EAT.

Predictor variables	Regression results					
	B	*β*	*t* value	*p* value	CI 95% for *B*	*ΔR* ^2^
*Regression #1*: BES predicting EAT						
Step #1: BES (*z*-score)	−2.83	−0.37	−10.92	**<0.001**	−3.34, −2.32	0.14
Step #2: BES squared	0.82	0.14	4.01	**<0.001**	0.42, 1.23	0.02
*Regression #2*: PBIAS predicting EAT						
Step #1: PBIAS (*z*-score)	−1.22	−0.16	−4.37	**<0.001**	−1.76, −0.67	0.025
Step #2: PBIAS squared	0.68	0.12	3.20	**0.001**	0.26, 1.10	0.013
*Regression #3*: BES × PBIAS predicting EAT						
Block #1:						0.152
BES (*z*-score)	−3.64	−0.47	−10.60	**<0.001**	−4.32, −2.88	
PBIAS (*z*-score)	1.21	0.16	3.50	**<0.001**	0.53, 1.88	
Step #2: BES × PBIAS interaction	0.66	0.10	2.90	**0.004**	0.21, 1.11	0.010

*B* represents the unstandardized regression coefficient. *β* reflects the standardized regression coefficient. Bold values refers to significance levels.

**Figure 2:**
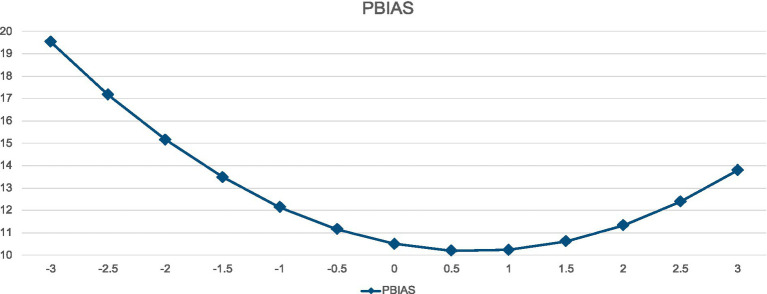
Curvilinear relationship between PBIAS and EAT.

Similarly, although the effect sizes were much smaller, there was a nonlinear relationship between PBIAS and EAT, as is also presented in Table 3 and Figure 2. As with BES, the most dramatic effects of PBIAS are in the lower ranges of the distribution, with the curve asymptoting as the values approach 0.5–1.0.

**Figure 3:**
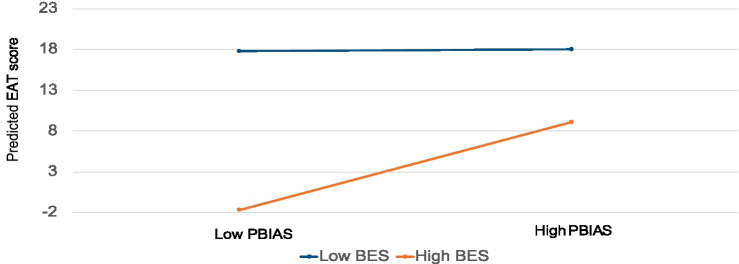
Interactions between BES and PBIAS predicting EAT.

Finally, we examined whether there was any moderation (interaction effect) between BES and PBIAS in predicting EAT scores. As you can see in Table 3 and Figure 3, there is a significant interaction effect where individuals with low BES (−2 SD below mean) have high EAT scores regardless of PBIAS scores, but where individuals have high BES scores (+2SD above mean), PBIAS influences EAT scores so that those who have high BES and low PBIAS scores have the lowest EAT scores, and those with high BES and high PBIAS have higher EAT scores. This more nuanced understanding of the nonlinear and moderated relationship of different attitudes within this population may enhance understanding of the phenomenon, and also to focus efforts at wellness where they may be most effective, particularly those with low BES.

## Discussion

According to the results of present study, significant relationships were identified between positive body image and eating attitude. The relationship between positive body image and eating attitudes has garnered significant attention in recent years due to the growing concern about body dissatisfaction and its potential impact on mental and physical well-being (Wardle and Beales, 1986; Lewis and Cachelin, 2001; Aruguete et al., 2004; Pedro et al., 2016; Prioreschi et al., 2017). All of past research is consistent with our study.

A study on Black and White College Students in United States showed there is a positive relationship between body image, and eating attitudes (Aruguete et al., 2004). Also, in a study on females in rural South African adolescents (Pedro et al., 2016), and South African young adult females (Prioreschi et al., 2017), researchers concluded that body image, eating attitudes, and BMI are significantly related to each other. In elderly women body image, body dissatisfaction, and eating attitudes are related (Lewis and Cachelin, 2001). Also, in a different age group, a study concluded that body image and food attitudes are significantly correlated in children (Wardle and Beales, 1986).

Positive body image refers to a subjective perception and acceptance of one’s own body, while eating attitudes encompass a range of behaviors and beliefs related to food consumption (Prioreschi et al., 2017). Individuals with positive body image tend to exhibit healthier eating attitudes. When people appreciate and accept their bodies, they are more likely to engage in mindful eating, prioritize nutritious foods, and avoid extreme dieting behaviors (Kichler and Crowther, 2009). Positive body image can act as a protective factor against the development of disordered eating patterns, such as binge eating or restrictive eating. Individuals with a positive self-image are less prone to succumb to societal pressures and unrealistic beauty standards (Rostampour et al., 2022). However, the media’s portrayal of idealized body images often contradicts realistic body diversity, leading to negative body image and contributes to unhealthy eating attitudes (Thomas et al., 2010).

Exposure to images of thin, flawless bodies can trigger feelings of inadequacy and discontent, potentially leading to disordered eating habits and a distorted relationship with food. The thin-ideal media culture can foster a desire to achieve an unrealistic body shape, thereby undermining positive body image and promoting a focus on external appearance over internal well-being (Costarelli et al., 2009; Thomas et al., 2010). Social comparison plays a pivotal role in shaping body image and eating attitudes. People often compare their bodies to those of others, leading to either positive reinforcement or increased dissatisfaction (Costarelli et al., 2009).

When individuals perceive themselves as less attractive their peers, they may adopt harmful eating behaviors in an attempt to conform to societal norms. Conversely, positive body image can serve as a buffer against the negative impact of social comparison, enabling individuals to appreciate their bodies despite perceived differences (Baceviciene and Jankauskiene, 2021). Cultural norms and societal values significantly influence both body image and eating attitudes.

Cultures that prioritize thinness as a beauty ideal are more likely to contribute to body dissatisfaction and disordered eating behaviors (Kichler and Crowther, 2009). Whereas, societies that celebrate diverse body types and emphasize holistic well-being, tend to foster healthier attitudes toward food and body image. The complex interplay between cultural influences, individual values, and personal experiences underscores the need for a nuanced understanding of the relationship between positive body image and eating attitudes (Lewis and Cachelin, 2001; Thomas et al., 2010).

Another main finding of the present study was the relationship between body esteem and eating attitude. The intricate interplay between body esteem and eating attitudes has been the focus of extensive research in the fields of psychology and health (Filaire et al., 2007). All of the cited research is in line with our study results that reported a significant relationship between these two variables. Body esteem refers to an individual’s overall evaluation and feelings about their own body, while eating attitudes encompass thoughts, beliefs, and behaviors related to food consumption (Filaire et al., 2007; Rouveix et al., 2007).

Low body esteem is in relation to negative eating attitudes. Negative body esteem can contribute to the adoption of harmful eating habits as a means of compensating for perceived physical shortcomings. This highlights the complex relationship between self-perception and eating behaviors. Social factors play a pivotal role in shaping both body esteem and eating attitudes (McVey et al., 2004). Peer comparisons, media portrayals, and societal beauty standards heavily influence how individuals perceive their bodies and approach food. Constant exposure to images of “ideal” body types can foster feelings of inadequacy and drive individuals to adopt unhealthy eating practices in an attempt to conform (Filaire et al., 2007). This suggests that societal pressures can contribute to a vicious cycle wherein poor body esteem fuels negative eating attitudes that in turn perpetuate low self-esteem.

Addressing the relationship between body esteem and eating attitudes necessitates a multifaceted approach. Interventions that promote body positivity, self-acceptance, and media literacy can help individuals develop a healthier relationship with their bodies and food (Trott et al., 2021). Encouraging a focus on overall well-being and cultivating self-worth beyond appearance can contribute to improved body esteem and healthier eating attitudes. Additionally, psychotherapy and counseling can offer support to individuals struggling with negative body image and disordered eating behaviors (McVey et al., 2004; Trott et al., 2021).

This study also showed modest effects of age and BMI in predicting eating attitudes. The relationship between age and eating attitudes is complex and influenced by biological, psychological, and social factors. In adolescence, societal pressures can contribute to body image concerns. Adulthood may bring a shift toward health-focused attitudes. Older age poses unique challenges related to health and societal perceptions (Aparicio-Martinez et al., 2019). Cultural and social influences shape attitudes across different age groups. Mental health and health considerations impact attitudes differently at various life stages. Individual differences play a significant role, and not everyone in a specific age group will share the same eating attitudes. Seeking support from healthcare professionals is crucial for those struggling with eating attitudes (Sari et al., 2021).

Time duration of social media usage was significantly (yet modestly) related with eating attitudes. Excessive social media use has been associated with negative effects on eating attitudes. Exposure to idealized body images, social comparison, cyberbullying, and the promotion of trends and diets can contribute to body dissatisfaction and unhealthy eating behaviors (Wilksch et al., 2020). The impact on self-esteem, the prevalence of filtered images, and the influence of social media influencers further play roles. Emotional well-being linked to social media use can also affect eating attitudes. It is essential to be mindful of these influences and foster a positive online environment to promote healthy attitudes toward food and body image (Saunders and Eaton, 2018).

Satisfaction on own sex had a significant relationship with eating attitude. Dissatisfaction with one’s own sex or body image can have a significant impact on eating attitudes. Individuals who are dissatisfied with their bodies may be more prone to developing unhealthy eating habits, such as restrictive diets, overeating, or engaging in disordered eating behaviors. These attitudes may stem from a desire to conform to perceived societal ideals or cope with negative emotions related to body dissatisfaction (Aparicio-Martinez et al., 2019).

## Limitations

These cross-sectional studies using self-report scales may have some important limitations. For example, we may not be able to trust the trustworthiness of responses. Participants might respond in a way they believe is socially acceptable rather than providing honest answers, particularly on sensitive topics like body image and eating attitudes. Also, convenience sampling method may limit the generalizability of the results.

This study also took place in the context of a very different sociocultural context than many others- and was primarily male, which may have implications for interpreting the results as western and European research has long focused on women and girls relationship to eating, body image, and body self-esteem.

There were two variables with significant missing data, which caused us to separate them out for analysis to preserve the sample size and power for the primary analysis. This is unfortunate but given the very modest effect sizes of these variables, should cause little concern as they were not the primary focus of this paper.

There may be unmeasured or uncontrolled confounding variables that affect the relationships between positive body image, body esteem, and eating attitudes. Factors such as socioeconomic status, access to healthcare, or mental health conditions could confound the results. Thus, these limitations may preclude the generalizability of our results of our findings.

## Implications

Understanding the complex interplay between positive body image, body esteem, and eating attitudes holds significant implications for various fields, including psychology, health, education, and media. This knowledge can inform interventions, policies, and strategies aimed at promoting holistic well-being and preventing the development of disordered eating behaviors.

1. Prevention of Disordered Eating Behaviors: Insights gained from studying these relationships can guide the development of prevention programs targeting disordered eating behaviors, including anorexia nervosa, bulimia nervosa, and binge eating disorder. Interventions that focus on cultivating positive body image and body esteem can help individuals build resilience against societal pressures and unrealistic beauty standards, thereby reducing the risk of adopting harmful eating attitudes.
2. Counseling and Therapy Approaches: Mental health professionals can use the insights gained from research in tailoring counseling and therapy approaches. Addressing body image concerns and promoting positive body esteem can become integral components of treatment plans for individuals struggling with eating disorders or body dysmorphic disorder. Therapeutic techniques can be designed to help individuals challenge distorted perceptions and develop healthier self-concepts.
3. Social and Cultural Change: Understanding the relationships among positive body image, body esteem, and eating attitudes can contribute to broader social and cultural change. Advocacy efforts can challenge unrealistic beauty standards and promote acceptance of diverse body types. This could lead to shifts in societal values, where well-being is prioritized over appearance, reducing the prevalence of body dissatisfaction and eating disorders.

## Conclusion

The research findings present the results of a multiple regression analysis that was performed to predict eating attitude based on various variables. The variables had a significant effect on the prediction of eating attitude were age, BMI, satisfaction with one’s sex, social media usage, positive body image, and body esteem. Body self-esteem was the predictor with the most impactful effect size in our analyses, and within a primarily male, non-western European culture. These findings may stimulate more thinking about these dynamics within other cultures and within male populations in addition to the long-standing interest in how female populations experience these dynamics.

These analyses also highlight the pervasive and potentially harmful impact of engaging with social media. While small effects, engagement with social media had a significant impact on attitudes toward eating, and continual exposure to individuals in crucial stages of development may have disproportionate impacts. We hope these findings stimulate further research into these and related issues.

## Data availability statement

The raw data supporting the conclusions of this article will be made available by the authors, without undue reservation.

## Ethics statement

The studies involving humans were approved by Ethics committee of Mazandaran University of Medical Sciences, Sari, Iran (Ethics code: IR.MAZUMS.REC.1402.520). The studies were conducted in accordance with the local legislation and institutional requirements. Written informed consent for participation in this study was provided by the participants’ legal guardians/next of kin.

## Author contributions

HS-N: Conceptualization, Supervision, Writing – original draft, Writing – review & editing. ES: Writing – original draft, Writing – review & editing. OG: Formal analysis, Writing – original draft. JO: Writing – original draft, Writing – review & editing, Formal analysis. AB: Writing – original draft, Writing – review & editing. AR: Conceptualization, Writing – original draft, Data curation. AG: Writing – original draft, Writing – review & editing, Conceptualization. OK: Writing – original draft, Writing – review & editing.
